# Proteins of the origin recognition complex (ORC) and DNA topoisomerases on mammalian chromatin

**DOI:** 10.1186/1471-2199-10-36

**Published:** 2009-04-28

**Authors:** Hong-gang Hu, Martina Baack, Rolf Knippers

**Affiliations:** 1 Department of Biology, University of Konstanz, D-78457 Konstanz, Germany; 2 Institute of Bioscience and Biotechnology, School of Science, Beijing Jiaotong University, 100044 Beijing, PR China

## Abstract

**Background:**

The process of DNA replication requires the separation of complementary DNA strands. In this process, the unwinding of circularly closed or long DNA duplices leads to torsional tensions which must be released by topoisomerases. So topoisomerases play an important role in DNA replication. In order to provide more information about topoisomerases in the initiation of mammalian replication, we investigated whether topoisomerases occur close to ORC in the chromatin of cultured human HeLa cells.

**Results:**

We have used different cell fractionation procedures, namely salt and nuclease treatment of isolated nuclei as well as formaldehyde-mediated cross-linking of chromatin, to investigate the distribution of topoisomerases and proteins of the origin recognition complex (ORC) in the chromatin of human HeLa cells. First we obtained no evidence for a physical interaction of either topoisomerase I or topoisomerase II with ORC. Then we found, however, that (Orc1-5) and topo II occurred together on chromatin fragments of 600 and more bp lengths. At last we showed that both topo II and Orc2 protein are enriched near the origin at the human *MCM4 *gene, and at least some of the topo II at the origin is active in proliferating HeLa cells. So taken together, topoisomerase II, but not topoisomerase I, is located close to ORC on chromatin.

**Conclusion:**

Topoisomerase II is more highly expressed than ORC proteins in mammalian cells, so only a small fraction of total chromatin-bound topoisomerase II was found in the vicinity of ORC. The precise position of topo II relative to ORC may differ among origins.

## Background

DNA replication requires the separation of complementary DNA strands. This process begins at the origin of replication under the direction of initiator proteins such as the T antigen of Simian Virus 40 (SV40) or the eukaryotic preinitiation complex including ORC (origin recognition complex). The unwinding of circularly closed or long DNA duplices leads to torsional tensions which must be released by topoisomerases. According to earlier biochemical work, high local concentrations of the eukaryotic type IB topoisomerase (topo I) and a type II topoisomerase (topo II) are required to release the torsional stress that accompanies the initiation and propagation of replication forks on closed circular SV40 viral DNA in vitro [1-3]. Further, topo I was shown to be mainly located in vivo at regions ahead of the replication forks on replicating SV40 DNA molecules, while topo II also occurs in pre-fork regions, but is essential for the decatenation of replicated DNA circles [4-7]. In fact, both eukaryotic topo I and topo II have the properties to release the positive supercoils that form ahead of the advancing replication forks, and the negative supercoils that accumulate in the replicated DNA sections [8,9].

In yeast cells, topo I seems to normally provide the swivel for replicative fork movement, but yeast mutants without topo I are viable because topo II can substitute for topo I in replication intermediates. However, topo II is absolutely required to decatenate linked chromosomes and to assist in the segregation of chromosomes at mitosis [8,9].

To provide additional information about topoisomerases in mammalian replication initiation, we investigated whether topoisomerases occur close to ORC in the chromatin of cultured human HeLa cells. ORC is the six-membered protein complex that marks the sites on eukaryotic chromosomes where prereplication complexes assemble, and where replication initiations occur at the beginning of S phase in the cell cycle [10,11]. Two major forms of ORC can be distinguished in asynchronously proliferating HeLa cells. One form, (Orc1-5), contains proteins Orc1p to Orc5p and is the predominant form in prereplicative G1 phase cells. During the S phase, protein Orc1p dissociates from ORC in HeLa cells leaving behind the second form of ORC, (Orc2-5). The sixth canonical Orc protein, Orc6p, appears to be loosely associated with either form and is usually lost during extractions or immunoprecipitations [12-15].

Mammalian cells express two isoforms of topo II, α and β, which are similar in their primary structures and enzymatic properties, but have different functions in proliferating cells. Topo IIα relaxes positive supercoils (as occur at replication forks) much more efficiently than negative supercoils [16]. Indeed, topo IIβ appears to have no preference for positive supercoils, and may be involved in the regulation of transcription rather than replication [17]. Therefore, we focus here on topo IIα (abbreviated below as topo II).

Recently, Abdurashidova et al. [18] have mapped the sites of active topo I and topo II at the mammalian lamin B2 origin using specific inhibitors that block the topoisomerase-reaction cycle after DNA strand cleavage. This elegant study provides evidence that both topo I and topo II are active at this particular origin at all phases of the cell cycle. However, the presence of the topoisomerases was determined indirectly by the footprints they leave in the form of DNA strand cleavages. The focus of the present study is different as it determines the presence of topoisomerases directly by Western blotting. We use different cell fractionation techniques which together show that topo II resides on chromatin in close neighborhood to ORC, but it became also clear that is only a small fraction of chromatin-bound topoisomerases, and that the vast majority of topo I and topo II resides elsewhere on chromatin.

## Methods

### Cell culture and cell fractionation

Human HeLa S3 cells were grown on plastic dishes in Dulbecco's modified Eagle's medium plus 5% calf serum. Nuclei were prepared and processed for salt extraction or treatment with micrococcal nuclease as described by Kreitz et al. [12]. Cross-linking by formaldehyde and the processing of cross-linked chromatin has been described in detail by Ladenburger et al. [19].

### Antibodies and immunoprecipitations

Antibodies against Orc and Mcm proteins were prepared in this laboratory and have been described before [12,20]. Monoclonal mouse antibodies against the DNA topoisomerases are from Biozol Diagnostica (Eching, Germany). Antibodies were used for immunoprecipitations and immunoblotting as in [20]. Ladenburger et al. [19] describe the conditions for chromatin-immunoprecipitation (ChIP) and for quantitative PCR including the primers used in this communication.

## Results

To investigate a possible association of topoisomerases with ORC, we first treated isolated nuclei from asynchronously proliferating HeLa cells with increasing salt concentrations and used the supernatants to perform immunoblottings for the determination of Orc1p, Orc2p, topo I and II.

As previously described [12], the (Orc2-5) form of ORC (see: Introduction) is released from isolated nuclei in buffers of 0.2 – 0.25 M salt; while the (Orc1-5) form is more tightly bound to chromatin and requires at least 0.32 M salt to be released from chromatin. We determined Orc1p as a proxy for (Orc1-5) and Orc2p (in the absence of Orc1p) for (Orc2-5).

**Figure 1:**
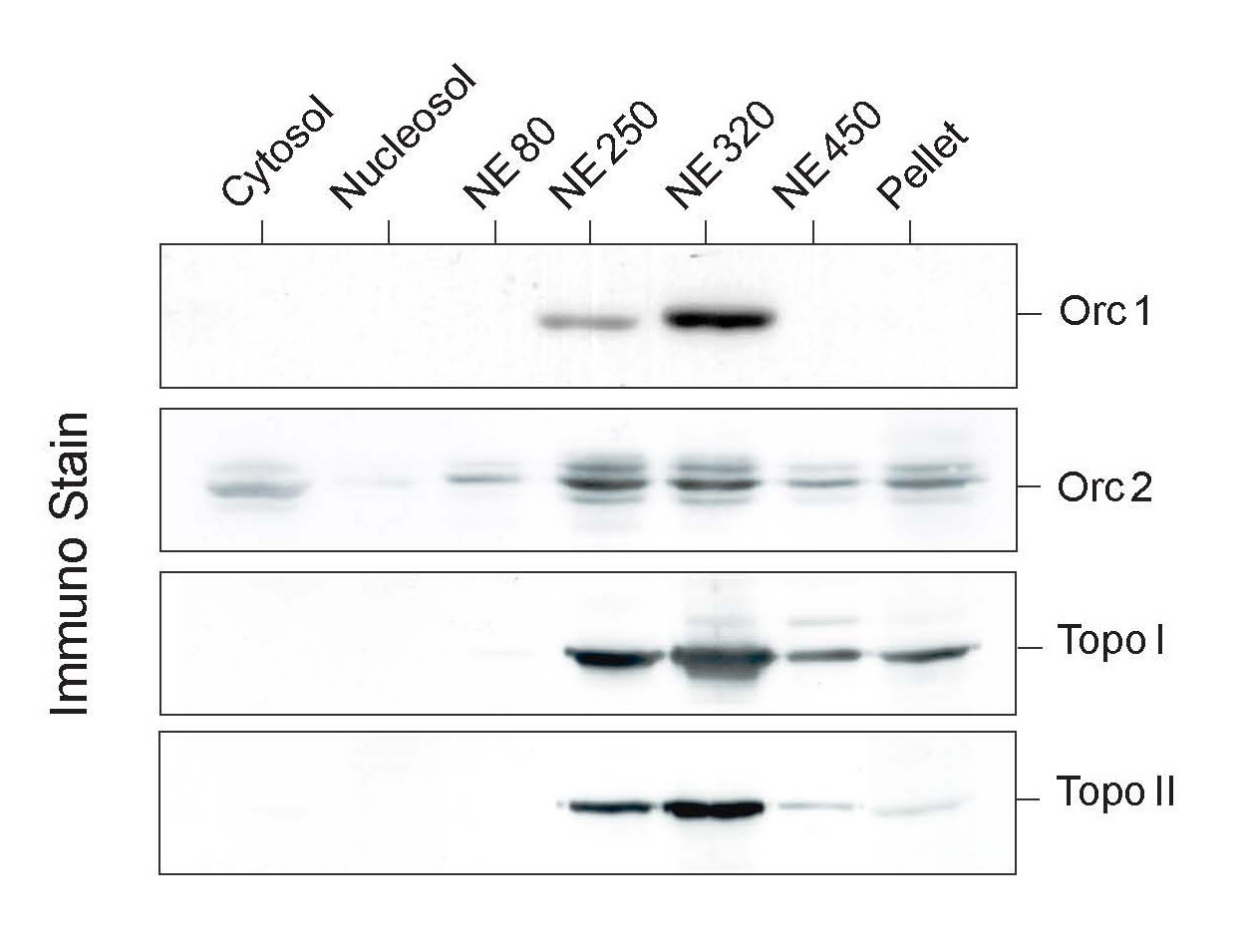
**Orc proteins dissociate together with topoisomerases from chromatin**. HeLa cell nuclei were prepared and treated with nuclear extraction (NE) buffer containing 80, 250, 320 and 450 mM NaCl as indicated. The supernatants were analyzed by polyacrylamide gel electrophoresis in the presence of SDS and western blotting.

The distribution of Orc2p and Orc1p over the various salt fractions in the experiment of Fig. 1 conforms to this description because most of Orc1p occurred in the high salt supernatant whereas Orc2p appeared in both, the 0.25 M and in the 0.32 M salt wash. In addition to its presence in the two ORC forms, small amounts of Orc2p were detected in the cytosol and in the insoluble pellet (Fig. 1) and may reflect the presence of Orc2p in centrosomes and heterochromatin, respectively, as recently described [21]. Both, topo I and topo II, were distributed in the fractions with 0.25 M and 0.32 M NaCl much like Orc1p (Fig. 1).

To determine whether the topoisomerases were in direct contact with ORC, we performed immunoprecipitations using specific antibodies against Orc1p, Orc2p, topo I or topo II. We were unable to detect coprecipitations of one or the other of the topoisomerases with either (Orc1-5) or (Orc2-5) (not shown) which makes it unlikely that the topoisomerases directly and stably interact with ORC under the experimental conditions.

**Figure 2:**
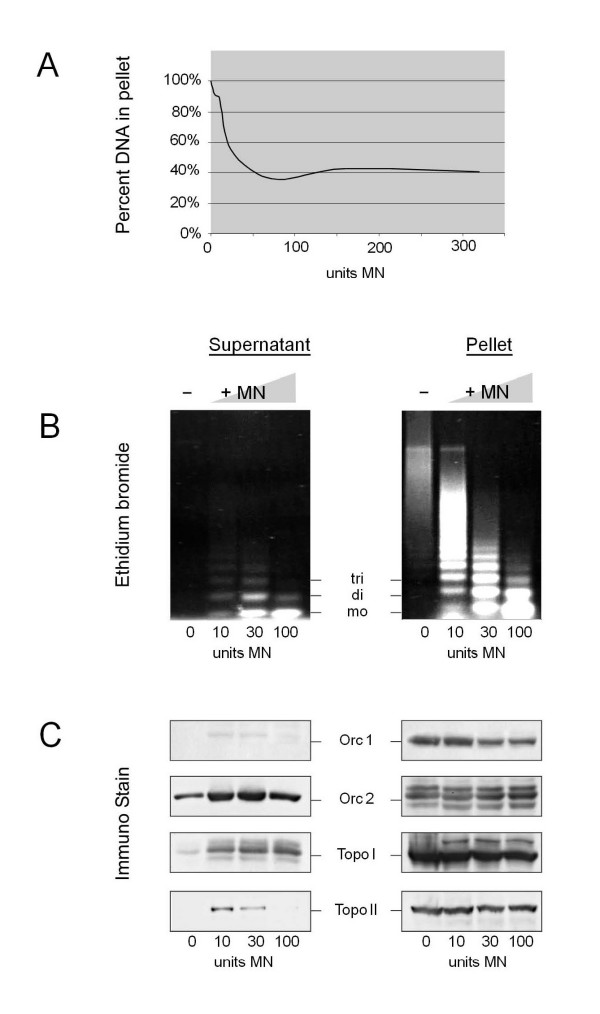
**Orc1p in (Orc1-5) together with a considerable part of topo I and topo II cannot be mobilized by nuclease treatment**. (A) Isolated nuclei (50 μg DNA) were treated for 15 min with MN in the amounts indicated. DNA concentrations were determined in the supernatants (not shown) and the pellet. (B) DNA in supernatants and the pellets were deproteinized and analyzed by agarose gel electrophoresis and staining with ethidium bromide. (C) Proteins in the supernatants and the pellets were investigated by western blotting.

This does not exclude the possibility that ORC and a topoisomerase are located at adjacent sites on the chromatin. To investigate this point, we treated isolated HeLa cell nuclei, which contain 50 μg DNA, with micrococcal nuclease (MN; 10 – 100 units/50 μg DNA) [12]. After an incubation time of 15 min with 10 units MN, 60 – 70% of the DNA in chromatin were mobilized and appeared in the supernatant of low speed centrifugation while the rest was insoluble in the Ca^++ ^– containing buffer needed for MN digestion. More units of MN (Fig. 2A) or longer incubation times (not shown) did not increase the fraction of solubilized DNA, but further degraded the DNA in both, the supernatant and the pellet fraction of chromatin (Fig. 2B). As expected from earlier work [12,15], we detected Orc2p as a component of (Orc2-5) bound to chromatin fragments in the MN supernatant whereas Orc1p in (Orc1-5) remained in the pellet fraction (Fig. 2C). Most of topo I and essentially all topo II were also in the pellet (Fig. 2C).

Based on previous experiments [19], we expected the (Orc1-5) complexes to be located at replication origins. To verify this prediction, we used quantitative PCR and found indeed that sequences corresponding to the mapped UPR origin [19] were 50 fold and sequences of the Lamin B2 origin [22] were 80 fold more abundant in the pellet fraction than origin-free control DNA (the exon 9 (Ex9) sequence in the human *MCM4 *gene [19]) (data not shown).

**Figure 3:**
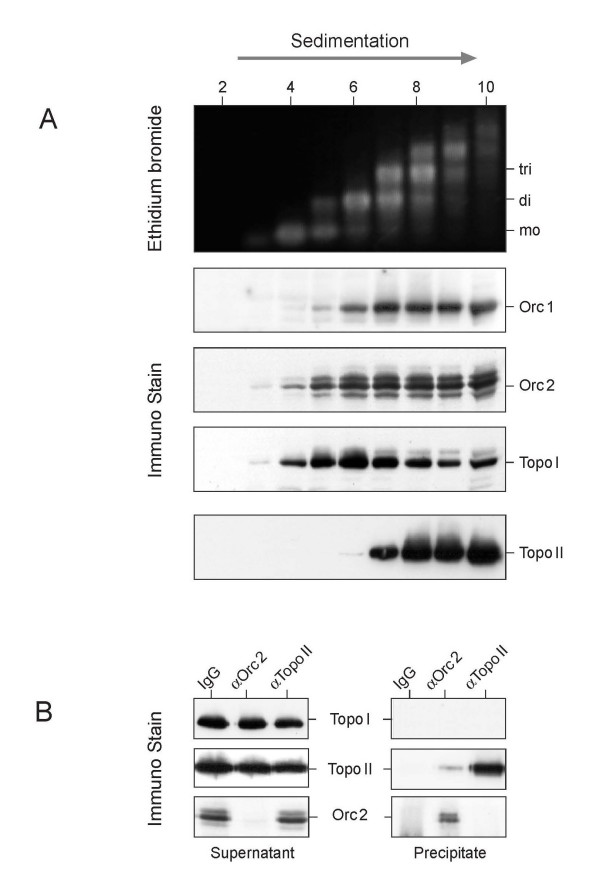
**Orc proteins cosediment mainly with topo II**. (A) The pelleted chromatin in MN-treated nuclei (Fig. 2) was partially suspended in a low salt – high EDTA buffer and investigated by centrifugation through a sucrose gradient (5 – 20%) made up in the same buffer. A part of each gradient fraction was deproteinized and analyzed by agarose gel electrophoresis and staining with ethidium bromide (upper panel). Another part of each fraction was investigated by western blotting. (B) The combined fractions 8 and 9 in (A) were incubated with either control antibodies (IgG) or antibodies specific for Orc2p or topo II. The precipitates were rigorously washed with 80 mM NaCl in NE buffer. The supernatants and the immunoprecipitates were analyzed by western blotting.

While the data in Fig. 2 showed that the two topoisomerases occurred together with (Orc1-5) in the chromatin pellet fraction (Fig. 2C), we wished to find out whether a topoisomerase and ORC colocalized on the same chromatin fragment. For this purpose, we took advantage of the fact that a considerable part of the chromatin pellet that remains after MN-treatment can be suspended in a low-salt, high-EDTA buffer (20 mM EDTA, 10 mM Hepes; pH 7.4) which does not disrupt protein-DNA interactions in chromatin. The resuspended chromatin fragments were separated by sucrose gradient centrifugation.

(Orc1-5) was expected to sediment in fractions 4 and 5 (sedimentation coefficient: 11 S) and free topoisomerases in fractions 2 and 3 (sedimentation coefficient: 3 – 5 S) of a sucrose gradient like that shown in Fig. 3A (data not shown). However, Orc1p and Orc2p sedimented faster and together with dinucleosomal and larger chromatin fragments. Topo I was distributed over many sucrose gradient fractions with a maximum that coincided with dinucleosomal fragments. In contrast, topo II sedimented faster and appeared together with Orc1p and Orc2p at chromatin fragments with three and more nucleosomes (Fig. 3A). To determine whether ORC and topo II occurred together on the chromatin fragments, we used the sucrose gradient fractions 8 and 9 (with trinucleosomal chromatin; Fig. 3A) for immunoprecipitations, and found that the Orc2p-antibodies precipitated, as expected, essentially all chromatin-bound Orc2p (compare supernatant and pellet in the immunoprecipitates of Fig. 3B) together with a small fraction of the immunologically detectable topo II, in contrast to topo I which could not be detected in the immunoprecipitates (Fig. 3B). This result suggests that (Orc1-5) and topo II occurred together on chromatin fragments of ca. 600 bp length.

However, the data also show that only a rather small fraction of total chromatin-bound topo II is linked to ORC, while the vast majority of topo II is distributed elsewhere in the chromatin. This may be the reason why in the experiment of Fig. 3B no Orc2p could be seen in the immunoprecipitate obtained with topo II-specific antibodies as these antibodies precipitated only part of the total chromatin-bound topo II. It seems that much of chromatin-bound topo II was inaccessible to the antibodies, and this could include the topo II in the vicinity of (Orc1-5).

**Figure 4:**
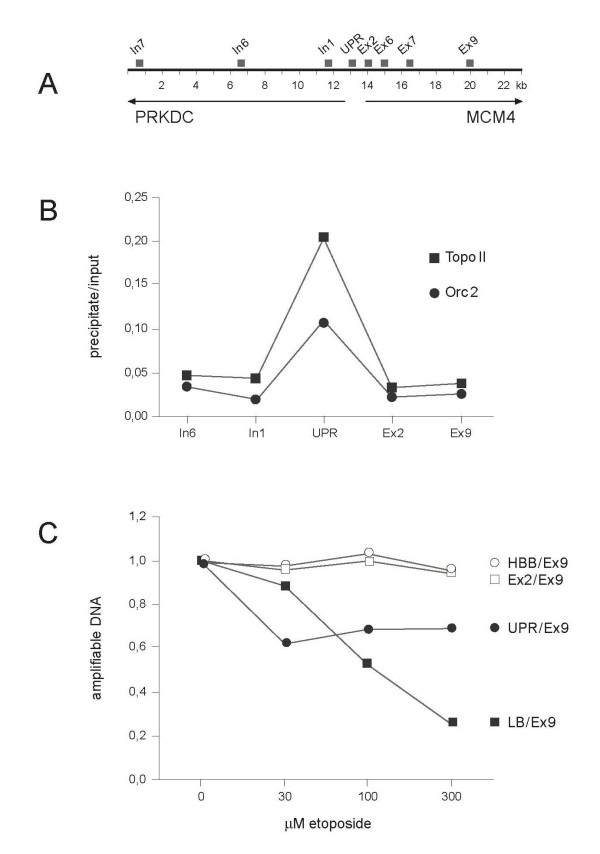
**Orc2p and topo II reside at the UPR origin**. (A) The UPR origin is located between the divergently transcribed human genes *PRKDC *and *MCM4*. Squares above the distance marker indicate the DNA regions analyzed by PCR. The data and the PCR primer are described in [19]. (B) The procedures for chromatin immunoprecipitation (ChIP) and quantitative PCR were decribed in detail [19]. Here we performed ChIP with Orc2p-specific (circles) and, in parallel, with topo II- specific (squares) antibodies. Quantitative PCR was performed with total deproteinized chromatin before precipitation (input) and the immunoprecipitate using the primers indicated in (A). (C) HeLa cells were treated for 90 min with the topo II-specific drug etoposide (from Sigma) in the concentrations indicated. The DNA was extracted and prepared for PCR. The data are expressed relative to the amplification of the exon-9 (Ex9) sequence which carries no topo II (see: panel B). Negative controls are the ratios of amplifiable DNA from exon 2 (Ex2; open square) in the *MCM4 *gene (see: panel B) and a sequence from an origin-distal part of human gene *HBB *[26](open circle). Positive control is the lamin B2 origin (LB; filled square) amplified using the primer pair described in [19]. UPR (filled circle) is the origin sequence in the *MCM4 *gene (see above: A and B).

We found it necessary to obtain additional evidence for a linkage of (Orc1-5) and topo II at replication origins. Therefore, we cross-linked chromatin proteins to DNA by a treatment of HeLa cells with formaldehyde exactly as described in [19]. Cross-linked chromatin was treated with MN producing DNA pieces of average lengths of ca. 500 bp. The chromatin fragments were immunoprecipitated with topo II- and Orc2p-specific antibodies, deproteinized and analyzed by quantitative PCR using primers specific for regions at and around the mapped origins of the human *MCM4 *gene (Fig. 4A). We found that both, topo II- and Orc2p-specific antibodies, enriched origin-proximal sequences relative to origin-distal sequences around the *MCM4 *gene (Fig. 4B). These data support the notion that ORC and topo II can simultaneously occur in a DNA region of several hundred bp at the UPR replication origin.

As additional evidence for the presence of topo II at the *MCM4 *origin, we used the topo II- specific drug etoposide which inhibits the topoisomerase reaction cycle after DNA strand cleavage [23]. Thus, if topo II is present and active, the MCM4 origin region should be broken in etoposide-treated cells and therefore inaccessible to PCR.

We found indeed that etoposide caused a decrease of amplifiable *MCM4 *origin DNA to 60 – 70% of the untreated control (Fig. 4C) indicating that at least some of the topo II at the origin is active in proliferating HeLa cells. As a control, we studied the lamin B2 origin in the same experiment and interestingly found that it is much more sensitive because etoposide-treatment reduced the amplifiable lamin B2 origin DNA to 20% of the control (Fig. 4C). This is in agreement with Abdurashidova et al. [18] who determined that topo II is active on the lamin B2 origin during all phases of the cell cycle.

## Discussion

We have used three different cell fractionation procedures to investigate the distribution of DNA topoisomerases relative to ORC on chromatin of proliferating human HeLa cells.

In a first procedure, we treated isolated nuclei with increasing salt distinguishing the prereplicative form of ORC, (Orc1-5), from its post-initiation form, (Orc2-5). The former needs higher salt concentrations than the latter for a dissociation from chromatin. Chromatin-bound topoisomerases I and II distribute almost equally between the two fractions, but no evidence for a direct interaction of one or the other topoisomerase with either ORC form was obtained through coimmunoprecipitation experiments. Thus, the cellular initiator proteins in ORC differ from the viral initiator T antigen which appears to physically interact with topoisomerase [3].

The second approach was digestion of chromatin with MN. Again, the pre- and the post-initiation ORC forms behave differently. The pre-initiation form (Orc1-5) is rather inaccessible to MN and remains in an unsoluble nucleoprotein fraction consisting of many components including architectural proteins and active transcription complexes [24]. In contrast, the post-initiation form (Orc2-5) is mobilized by MN just as the majority of chromatin. The topoisomerases distribute between the MN-accessible and the insoluble fraction. Analysis of the pellet fraction by sucrose gradient centrifugation helped to clarify the situation as most of topo I sedimented in gradient fractions without ORC whereas topo II cosedimented with and were probably bound to larger chromatin fragments that also carried ORC. Indeed, immunoprecipitations directly showed that (Orc1-5) and topo II occurred on the same chromatin fragments of 600 and more bp lengths.

Our third method to study the binding of ORC and topoisomerase to chromatin was formaldehyde-mediated cross-linking. We have used this technique before in our studies on the chromatin-binding of ORC and Mcm proteins [19,20]. Here, we show that ORC and topo II (but not topo I) are located together on chromatin fragments of ca. 500 bp.

## Conclusion

Taken together, the data reveal that only a small fraction of total chromatin-bound topoisomerases are located in the neighborhood of ORC, whereas the vast majority is located elsewhere in the chromatin. In particular, we have no evidence that topo I occurs close enough to ORC on chromatin to be coprecipitated with ORC on MN digestion products, or to be cross-linked together with ORC on chromatin fragments of ca. 500 bp. In this context, we note a recent publication showing that it is topo II, not topo I, which is most active relaxing torsional strain on nucleosomal DNA [25].

Indeed, topo II could be detected in the vicinity of ORC. Topo II is expressed in a large excess over the ten thousand or so ORC molecules per HeLa cell nucleus [12], and therefore the great majority of topo II must be on chromatin sites other than replication origins. However, it is interesting that in our cross-linking experiments topo II was found to be enriched in the origin/promoter region of the *MCM4 *gene (and of the *TOP1 *gene; not shown) but not in the transcribed sections. This clearly indicates that topo II does not randomly distribute on the genome, but prefers specific sites of which replication origins are an example. We cannot say yet whether topo II and ORC reside so closely on chromatin as to touch each other as the work of Abdurashidova et al. [18] seems to suggest. We like to argue though that the precise positions of topo II may differ among origins. This may be the reason why we found the lamin B2 origin to be more sensitive to the topo II- poison etoposide than the *MCM4 *or *TOP1 *origins. The position of topoisomerases may depend on other genetic elements in the vicinity of the origins such as overlapping enhancer or promoter elements. However, this point requires additional attention.

## Authors' contributions

HH participated in carrying out the study, ChIP assay, quantitative PCR and in drafting the manuscript. MB participated carrying out the study, cell fractionation, immunoprecipitation, PCR and in drafting the manuscript. RK participated in conceiving the study, study design and in drafting the manuscript. All authors read and approved the final manuscript.
